# Machine learning meets partner matching: Predicting the future relationship quality based on personality traits

**DOI:** 10.1371/journal.pone.0213569

**Published:** 2019-03-21

**Authors:** Inga Großmann, André Hottung, Artus Krohn-Grimberghe

**Affiliations:** 1 HMKW Hochschule für Medien, Kommunikation und Wirtschaft, University of Applied Science, Berlin, Germany; 2 LYTiQ GmbH, Germany & Indian Institute of Information Technology Allahabad, Prayagraj, India; 3 Department of Business Information Systems, University of Paderborn, Paderborn, Germany; Western Sydney University, AUSTRALIA

## Abstract

To what extent is it possible to use machine learning to predict the outcome of a relationship, based on the personality of both partners? In the present study, relationship satisfaction, conflicts, and separation (intents) of 192 partners four years after the completion of questionnaires concerning their personality traits was predicted. A 10x10-fold cross-validation was used to ensure that the results of the linear regression models are reproducible. The findings indicate that machine learning techniques can improve the prediction of relationship quality (37% of variance explained), and that the perceived relationship quality of a partner is mostly dependent on his or her own individual personality traits. Additionally, the influences of different sets of variables on predictions are shown: partner and similarity effects did not incrementally predict relationship quality beyond actor effects and general personality traits predicted relationship quality less strongly than relationship-related personality.

## 1. Introduction

For many adults, it is a central goal in life to attain and to maintain a satisfying romantic relationship, which plays a key role in fostering well-being [1]. A review by Kiecolt-Glaser & Newton [2] and a meta-analysis by Proulx, Helms, & Buehler [3] showed moderate cross sectional and longitudinal correlations of RQ (relationship quality) to physical and mental health. But why are some relationships successful and satisfying while others even have a negative impact on physical health? A study by Solomon & Jackson [4] using a representative, longitudinal sample suggested that the personality of a couple influences the overall relationship satisfaction, which in turn influences the likelihood of break-up. Because most personality traits are stable across different relationships, this naturally leads to the question if they can be used to predict the RQ of a possible future couple. This could allow for forms of matchmaking which increase RQ and therefore the wellbeing of both partners.

### 1.1. Reproducible success of previous prediction models

Existing research has already addressed the question of to what extent it is possible to predict RQ based on personality. However, previous approaches working with similarity, actor and partner variables mostly used a simple correlational approach, e.g. derived from structural equation-based modelling and generally found only modest effects [5, 6]. Some approaches using mathematically more sophisticated models optimised predictive replicative power for break-up [7] based on characteristics of marital interaction in a present partnership such as communication, conflict, and mood variables [8]. For example, an accurate model was developed with 10-fold CV (cross-validation) and with discriminant analysis by the test system ENRICH. It predicted break-up with a longitudinal accuracy of 80–90% [9] but only works properly for existing relationships. Methods that are based exclusively on the highly stable personality traits of the partners could, in contrast, also be used to predict the RQ of a possible future couple. However, until now, the question is left open if personality traits not only reproducibly predict initial romantic attraction—as a very early aspect of RQ—in a cross-validational design [10] but also later RQ.

Recent work has shown that ML (machine learning) methods could contribute to solving the problem of the reproducibility of a researcher’s analysis [11]. Traditional methods of analysing data in the field of psychology follow an explanatory pattern. This leads to issues such as overfitting of the evaluation procedure to specific data sets [12, 13]. ‘P-hacking’ [14] or less tendentiously, data-contingent analysis [15] is one of the most common causes of overfitting biases in psychological research and is especially relevant for small, non-representative data sets. Yarkoni & Westfall [11] have discussed that a short-term emphasis on reproducible prediction could ultimately improve the ability to explain the causes of behaviour in the long term and therefore increase theoretical understanding.

### 1.2. Actor-, partner- and similarity effects

To which extent certain character traits are linked to RQ has also already been addressed in preceding research. For the Big Five, higher actor than partner effects–as well as no, or only very slight, additional effects of partner similarities–for RQ prediction were reported: in three very large nationally representative samples of married couples from Australia, the United Kingdom, and Germany, actor effects accounted for approximately 6% of the variance in relationship satisfaction, while partner effects explained 1% to 3% and similarity effects less than 0.5%, respectively after controlling for actor and partner effects [16]. Studies on the incremental effects of similarity regarding attitudes, values, life goals, and other traits have so far been inconsistent. In some countries, additional minor effects were found, e.g. in a large German study predicting a break-up after one year [17] and in two nationally representative Chinese studies predicting relationship satisfaction [18]. In contrast, two representative Dutch studies did not find a significant additional effect of similarity [19].

### 1.3. Effects of relationship-related and general personality

Relatively consistently across existing studies, relationship-related personality traits accounting for attachment and love styles have been found to be slightly more related to RQ than more general personality traits [20]. Traits associated to a general competency in relationships as secure vs. insecure attachment style turned out to be the most important for RQ. More general personality traits only slightly affected RQ: a meta-analysis [21] as well as a cross-cultural study on representative samples from Australia, the UK, and Germany [16] showed that scores of four of the five-factor model personality factors correlated positively with the level of relationship satisfaction for the actor and the partner. The strongest associations were found for agreeableness and emotional stability, followed by conscientiousness, and then extraversion. No consistent gender effects occurred. For openness to experience, results were not consistent. So far, an open research question remains if general or relationship-related traits have an incremental validity for longitudinal RQ prediction. They might not, because they share common variance concerning the part of personality which is relevant to social interactions.

### 1.4. The present study

**Fig 1:**
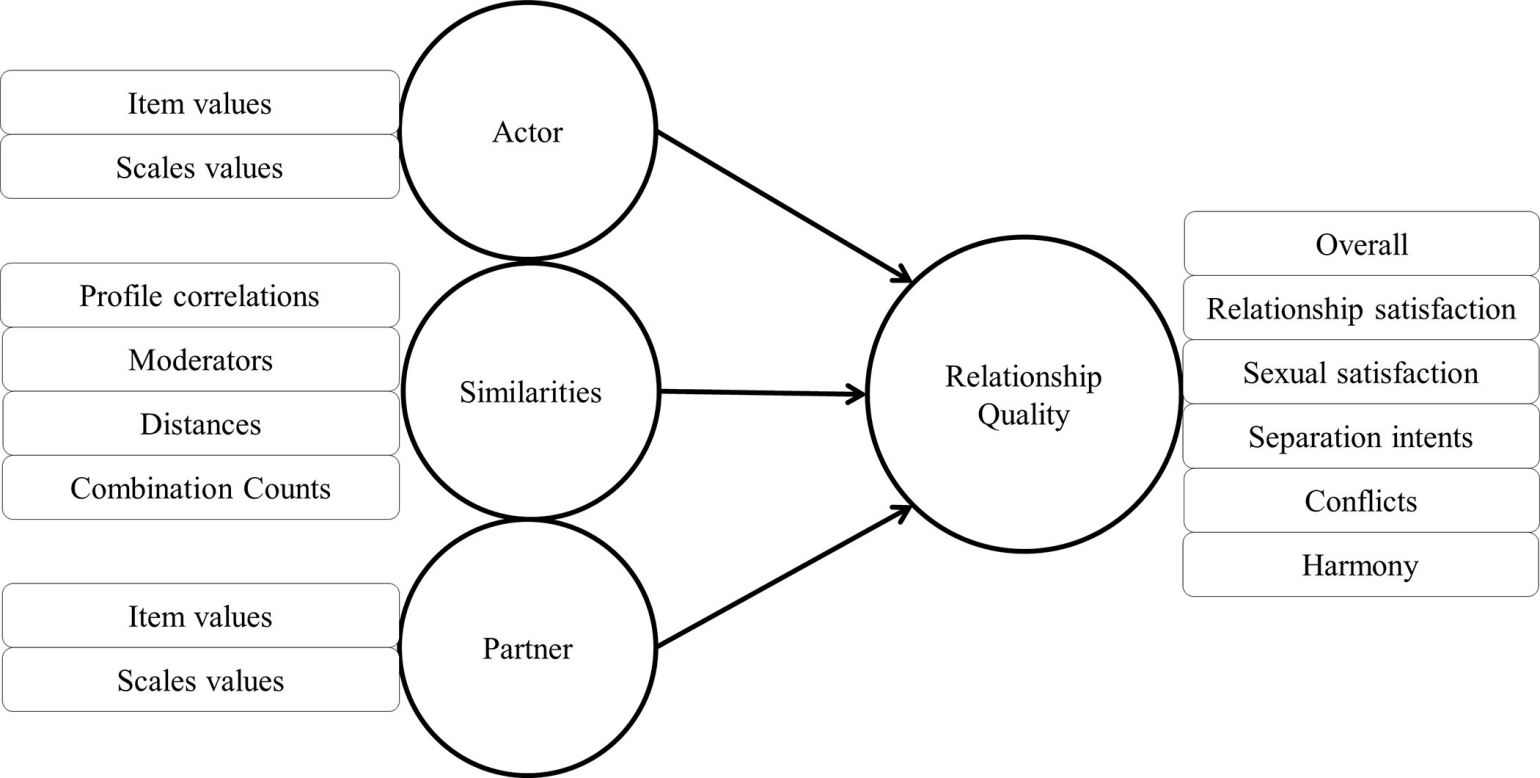
Linear regression model to predict RQ using personality variables. Different RQ measures on the right were predicted by different sets of personality scores on the left. CC: Combination Counts. RQ: Relationship Quality.

Following a recent methodological trend in the field of cognitive and social psychology, we applied classic methods from the ML literature [22–25], e.g. to deal with the characteristics of the given dataset, namely a large number of highly correlated variables and a small sample size [11]. In a prior cross-sectional analysis of couple’s personality data, the results of RQ prediction based on ML correspond with those of previous research on large datasets while outperforming these in the predictive effect sizes [26]. In the present study we use the same analysis methods and partly the same dataset. The current work is the first attempt to tackle longitudinal RQ prediction based on self-assessed personality traits using ML methods. The following variables (Fig 1, sets of variables, left) are used to develop (train) and cross-validate (test) the models which predict RQ (Fig 1, RQ measures, right).

Our analyses with linear regression models have the three following sub-focuses: (1) Reproducible predictive power: We evaluated how much variance of the overall continuous RQ ML-based models trained on all variables can explain and how these compare to the success of simpler correlation-based approaches of former studies. (2) Actor-, partner- and similarity effects: In ML-based models, actor, partner, and similarity variables were tested for incremental effects in predicting RQ over and beyond one another–as conducted in some prior studies using traditional regression models. (3) Relationship-related and general personality: (3a) Relationship-related and general personality traits were tested for incremental effects in predicting RQ over and beyond one another. (3b) Models based on only conflict-, value-, sex-, love- or interest-related variables and models based on variables of only agreeableness, emotional stability, conscientiousness, extraversion or openness were tested for their predictive performance and compared with one another. Conclusions about different domains sharing relevant parts of the relationship-related personality were made.

## 2. Materials and method

### 2.1. Operationalisation

In a longitudinal design, personality is measured at time 1 (T1) and RQ is measured at time 2 four years later (T2). T1 data is partly identical with the prior cross-sectional study [26].

#### 2.1.1. T1 personality

The testing of personality traits corresponds with the one used in the cross-sectional analysis [26]. Personality characteristics were measured with the help of questionnaires for self-assessment—as is common in online dating (Table 1). Contents of Items contain statements about former experiences in close romantic relationships but do not refer to a specific partner. The answers scale ranges from 1 to 5:

- 1 as “completely false”,
- 2 as “more false than true”,
- 3 as “part-part”,
- 4 as “more true than false”,
- 5 as “completely true”.

**Table 1:** Operationalization of personality variables at T1 with content domains.

Personality	General personality	Relationship-related personality
Questionnaire	Personality Domain Inventory PD-I [26]	Bonding- and Relationship Personality—Inventory BBP-I [28]
Items	323 items from construction pool	678 items from construction pool
Domains	• Agreeableness, emphasis on emotion and warmth • Pro-sociality, helpfulness, and empathy • Risk appetite, thirst for adventure, sportiness • Neuroticism, fearfulness, insecurity • Extraversion, gregariousness, enterprise • Conscientiousness, reliability, orderliness • Will to achieve, assignment, ambition • Aggressiveness, trouble tendency, hostility • Openness to experiences, creative tendencies • Intelligence, mental efficiency • Spirit of research, will to experiment, interest in technology	• Sexuality, adventure, and desire • Allurement, charm, and attractiveness • Market-orientation and pride • Dominance, disputability, and aggressiveness • Unsureness, doubt, and disappointment • Love, erotic behaviour, and understanding • Troth, morals, and stability • Bond, commitment, need for nearness, dependency

Notes. Reprinted from Großmann, Hottung, & Krohn-Grimberghe [26].

The 229 facets consist of 5 to 10 very homogeneous items and correspond with the original, rationally designed scales of the Personality Domain Inventory [27] and the Attachment- and Relationship-related Personality—Inventory [28]. All Person correlations to RQ as well as the descriptive statistics are presented on our open source page. A large number of homogeneous facets instead of a little number of heterogeneous domains that include correlating facets to allow a differentiated variable selection was analysed.

Each item and scale can be classified as

- an actor, a partner, or a similarity variable
- a relationship-related personality or a general personality variable.

Furthermore, some of the scales can be classified as

- indicator of emotional stability, extraversion, openness, agreeableness, or conscientiousness
- love-related, interest-related, sex-related, conflict-related, or value-related contents

#### 2.3.1. T1 similarity

Similarities were calculated using three different scores:

1. Distances: Similarity scales were calculated by adding up the distances between the two partners item responses. Additionally, item distances between items of both partners are added as variables.
2. Moderators: Moderators were calculated for each scale by z-value scale partner 1 multiplied by z-value scale partner 2.
3. Combination counts: Different combinations of item values were quantified in scores that count different combinations of actors and partners values for the same items of a scale. (Dis-)similarity combination counts emerge from combinations of low and high item values of both partners.

#### 2.1.2. T2 relationship quality (RQ)

Relationship happiness and relationship stability are generally evaluated as main components of RQ [29, 30]. Relationship happiness is measured by perceived relationship satisfaction, sexual satisfaction, conflicts, and harmony in different domains. Stability is measured by separation intents and actual break-ups. The common diagnostic instruments used to measure these aspects of RQ at T2 are described in Table 2. The average of these scales was used as a measure for the general RQ (called RQ overall). Since the perceived RQ can vary between the partners of a couple, all RQ measures were determined for each of the partners individually.

**Table 2:** Self-assessed aspects of RQ measures.

Questionnaire	Contents (nb. of items)	Scaling	RQ measures
Questionnaire for partnership diagnostics FDP [31]	Amount, intensity, duration and negativity of conflicts (4), perceived constrictions due to current partnership (1)	1 none to 6 high	Conflicts
	Overall satisfaction in and with current partnership (2)	1 very dissatisfied to 7 very satisfied	Separation intents
Life Satisfaction Questionnaire FLZ [32]	Satisfaction with sub-aspects of life domain sexuality (7)	1 very dissatisfied to 7 very satisfied	Sexual satisfaction
	Satisfaction with sub-aspects sub- aspects of life domain partnership (7)		Relationship satisfaction
Marital Satisfaction- Inventory-Revised MSI-R [33], Dyadic Adjustment Scale DAS [34], Questionnaire for partnership diagnostics FDP [31]	Harmony in main domains within partnerships (25) including: relationship notions, problem solving, arrangement in corporate, basic and future domains, positive emotions	1 none to 5 high	Harmony
Marital status inventory MSI [35]	Separation intents (1), Break-up (1, dichotomous)	thoughts about dissolution: 0 no, 1 seldom, 2 often, 3 thoughts become intents, 4 concrete separation intents, 5 serious plan to break-up, 6 plan already began to implement, 7 broke up	
Averages of all z-standardised above scales–which were polarised into the same direction		z-value	RQ overall

### 2.2. Couple data

#### 2.2.1. Sample description

The whole longitudinal sample consists of N = 192 heterosexual German individuals who were mostly adults with above-average educational levels and living in short or long-term relationships at T1. Overall, the sample consists of (1) 110 partners of 55 couples both completing T1 questionnaires about personality and T2 questions about RQ and (2) 82 partners of 82 couples from which only one partner completed personality questionnaires at T1 and questions about RQ at T2. Individuals who participated in the T1, but not in the T2 assessment were treated as drop-outs. However, their personality data from T1 was of course used to predict their partners RQ if the latter took part in T2 (being the case for n = 82).

The median relationship duration was Med. = 41 with SD = 116.5 months (Range: 1–519). 80 participants (41.7%) had a university degree, 61 (32.3%) had a high-school diploma (German: *Abitur*), 35 (18.2%) had finished secondary education and 10 (1.92%) had a lower set of qualifications. six participants did not state their level of education. 74 (38.5%) had no children, 30 (15.6%) had one, 54 (28.1%) had two and 26 (13.5%) had more than two (maximum = 6). Profile correlations of partners for relationship-related personality (Mn. = .487, SD = .165, ν = .335, SE = .178, n = 192) and for general personality (Mn. = .346, SD = .173, ν = -.914, SE = .194, n = 157) are moderate. From 192 partners tested at T2, 55 broke up while 137 were still a couple at T2.

#### 2.2.2. Patchwork dataset

**Fig 2:**
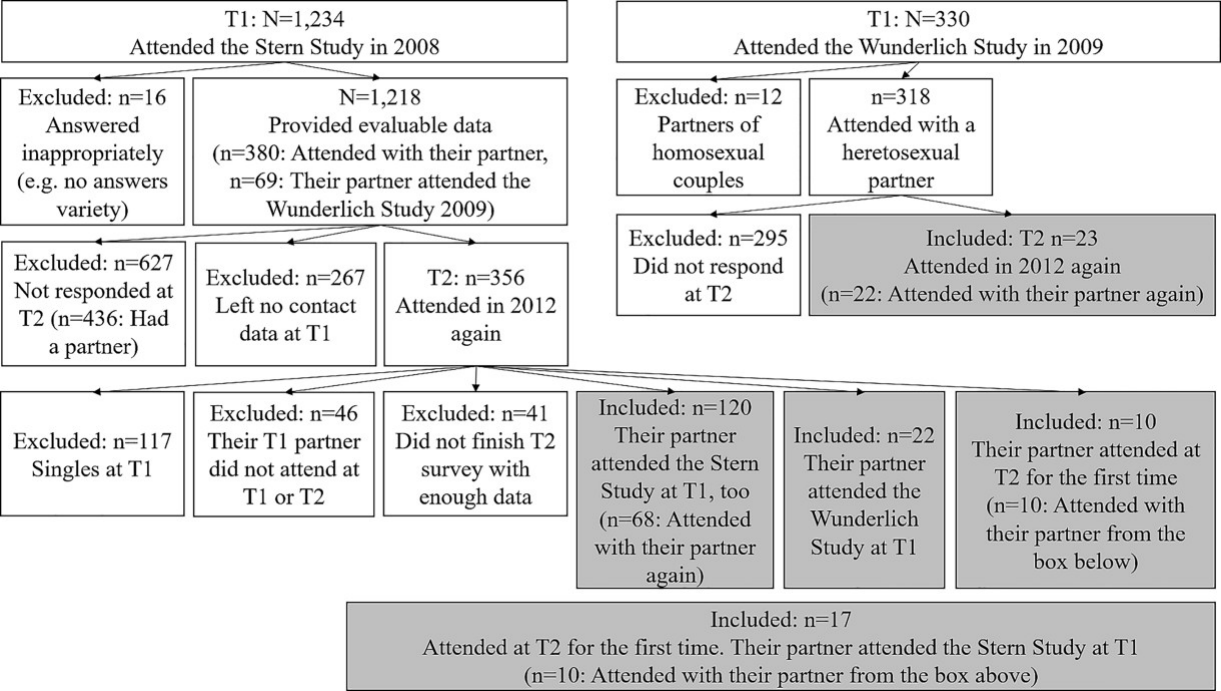
The figure depicts the exclusion criteria and the number of participants affected by each (if not already excluded for a preceding reason). **Participant flow.** The figure shows that the main data source of the 192 partners used in the current study were the 120 partners who took part in the Stern study as well as in the follow-up four years later. The included study subjects are marked in grey. No information on the drop-out due to starting but not finishing the T1 surveys could be found.

The participant flow and exclusion criteria are shown in Fig 2.

T2 data was measured in an online survey at the University of Hamburg in Germany. For T1, we work with a patchwork data set of couple’s data for individuals:

- n = 380: Both partners’ personalities at T1 were completed as part of a survey which recruited through an article in the German magazine Stern [28], n = 120 of these provided T2 RQ data and were therefore used in the described sample.
- n = 27: Partner 1 participated in the Stern study at T1 but without their partner, who only provided T2 data. In these cases, personality data of partner 2 was used from T2 and personality data of partner 1 from T1. In all other subsamples, personality data was used from T1 only.
- n = 69: One or both partners did not take part in the Stern study, but in another follow-up study one year later [36], n = 45 of them provided T2 RQ data. Only the last mentioned were used in the described sample.

The personality data of the partners who participated at T1 in the Stern study (n = 380+27 = 407) were used for cross-sectional predictions in the pilot study [26]. At T2, 147 of these 407 partners participated. Describing the dataset overlap, the T1 personality data from these 147 was used for the longitudinal predictions in the current study as well.

#### 2.2.3. Missing data

Mainly, at T1 n = 124 (64.6% of sample) are lacking less than 10% of the 4,904 personality variables, n = 22 (11.5% of sample) do not include more than 31.4% and no one is missing more than 54.3%. The missing values occur because only the *Stern* study collected the whole item pool. Missing values were replaced by the mean: for further explanation see section 2.3.2.

#### 2.2.4. Ethical evaluation

Since the present study does not include any questionable ethical elements, we did not seek approval of an ethics committee/IRB: Our study in the field of social sciences exceptionally involved consented adults who have no other advantage from their participation than a good feeling to contribute to research and an individual feedback on their personality traits. No element of coercion was involved and participants were informed about the details of the study. Furthermore, the experiment is an evaluation which does not include an intervention. Only Non-invasive research methods are applied, i.e. attendees just fill out questionnaires. The personal data was completely self-observed and processed anonymously.

### 2.3. Procedure

The ML-based evaluation is closely following the procedure described in Großmann, Hottung, & Krohn-Grimberghe [26]. For a detailed introduction to machine learning we refer to James et al. [37].

After the z-standardization of all variables elastic net models were trained and evaluated in a CV setup. This process was repeated using different variable groups as model input as well as different RQ measures as model output to allow for a detailed comparison. The predictions of the models were evaluated using the mean squared error (MSE) and the coefficient of determination (r^2^).

We evaluated different methods to reduce the number of variables (e.g., by predicting based on scale facets only, or based on scale facets in addition to item values) but we could not find any noticeable impact of these methods on the results. Therefore, we just present the results for all available item and scale variables here. In the following, we describe the used elastic net regression and the model evaluation in more detail.

#### 2.3.1. Elastic net regression

Elastic net regression is especially well suited for data sets with small samples and a large number of correlated variables [11]. For a detailed description of elastic net we refer to Hui & Hastie [38].

Elastic net regression optimises the weight vector ***w*** of a linear regression model ($\hat{y}=w_{0}+x_{1}w_{1}+\ldots +x_{p}w_{p}$, with ***x***_**1**_,…,***x***_***p***_ being the variable vector) under consideration of two linearly combined regularisation terms:

$$argmin_{w}(\frac{1}{2*n}*\|y-Xw{\|}_{2}^{2}+\alpha *\lambda *\|w{\|}_{1}+0.5*\alpha *(1-\lambda )*\|w{\|}_{2}^{2})$$

where n is the number of samples, y is the target value vector and X is the variable matrix. Alpha is used to set the degree of regularization and lambda defines the ratio of the two regularisation terms ‖*w*‖_1_ and $\|w{\|}_{2}^{2}$, where ‖*w*‖_1_ is the lasso penalty and $\|w{\|}_{2}^{2}$ is the ridge penalty. Lambda was set to *λ* = 0.5 while the selection of alpha was incorporated into CV procedure (using a nested CV as described by Cawley et al. [39]). During a preliminary evaluation we noticed a positive impact of tuning alpha but not of tuning lambda compared to fixing it (to *λ* = 0.5). Since hyper-parameter tuning in a nested CV setup is very computationally intensive (even for small datasets), we only focused on tuning the parameter alpha which sets the overall degree of regularization to prevent an overfitting of the models.

#### 2.3.2. Cross-validation

We used a repeated 10-fold CV setup for the evaluation of the elastic net models. For a more detailed description of the applied cross-validation procedure we again refer to Großmann et al. [26].

The dataset is split into 10 roughly equally sized folds. Each fold is used once (as a test set) to evaluate the prediction quality of a model that was trained on all other remaining 9 folds. Thus, a model is never evaluated on the data that was used for its training. This is of particular importance, because the small size and the high number of variables lead to a high risk of overfitting. To further enhance the reproducibility of the results the described process is repeated ten times (each time with different splits for the CV folds) as recommended in Bouckaert & Frank [40]. The overall performance is then given by the average performance of the models on the different test sets.

#### 2.3.3. Evaluation Measures

To evaluate the quality of the predictions MSE and r^2^ were used. Please note that r^2^ can be negative if model training and model evaluation are performed on different datasets (as it is the case here), because the predictions can be worse than the average target value of the test set, which consequently results in a negative r^2^ value.

For the evaluation of the statistical significance of the results the corrected resampled t-test was used. It is especially suited for the evaluation of results generated with a repeated CV [40], where the same data is used in multiple CV iterations.

#### 2.3.4. Handling of dyadic and missing data

The dyadic nature of the data (i.e., the responses of the two partners of couple are not independent) was taken into account to avoid distortions by dependency. Both partners of a couple were either both in the training set or both in the test set for all CV iterations. This ensures that the test set does not contain entries that are dependent on entries in the training set, which could lead to biased performance estimates.

The applied elastic net regression requires a dataset without missing values: Thus, missing values were replaced by the mean of the non-missing values prior to model training. To ensure that no information from the test set leaks into the training set (which would bias the results) the mean was calculated only based on the training set as part of the CV procedure (in contrast to calculating the mean based on the whole dataset outside of the CV procedure). The calculated mean was then used to replace missing values in training and test set.

## 3. Results

### 3.1. Descriptive statistics RQ measures

Table 3 shows descriptive statistics of the RQ measures and their inter-correlation. Pearson correlations between the different RQ measures were generally positive and ranging from low to high (.85> r >.15). RQ measures were positively correlated (.8> r >.5) between partners.

**Table 3:** Descriptive statistics about RQ (n = 192 for T2).

RQ measures	Mn	SD	CA	P1P2 r	1	2	3	4	5	6	7
1 T2 Harmony	3.78	.691	.944	.760***							
2 T2 Conflicts	3.38	1.16	.856	.583***	.539***						
3 T2 Relationship satisfaction	5.43	1.28	.897	.652***	.834***	.505***					
4 T2 Sexual satisfaction	5.14	1.46	.909	.651***	.504***	.227**	.572***				
5 T2 Separation intents	.242	.954	.892	.520***	.786***	.474***	.791***	.264***			
6 T1 n = 476 Separation intents	476	.484	.821	.632***	.385***	.302***	.371***	.264***	.362***		
7 T2 Break-up				1.00***	.457***	.296***	.387***	.164*	.517***	.293***	

Notes. r P1-P2 = Intra-couple Pearson correlation. SD: standard deviation. CA: Cronbachs alpha.

* p < .05

**p < .01

***p < .001.

### 3.2. Model performance

Similar to [26], we used a resampled CV set-up in combination with an appropriately modified t-test for the baseline comparison to ensure that our results are reproducible and valid despite the small sample. We omit the reporting of confidence or credibility intervals because they are not suited for a proper evaluation of results based on repeated CV [41]. For comparison, the baseline is defined as the performance of a model always predicting the average value of the according RQ measure. Table 4 presents the predictive performance of models using different combinations of actor, partner, similarity, personality, and domain variables.

**Table 4:** 10*10-fold CV performance of the elastic net models based on different variable sets (n = 192).

nb. of	variables	RQ overall		Separation intents		Relationship satisfaction		Sexual satisfaction		Conflicts		Harmony overall	
		MSE	r^2^	MSE	r^2^	MSE	r^2^	MSE	r^2^	MSE	r^2^	MSE	r^2^
	baseline	1.00	-.12	.92	-.14	1.00	-.13	1.03	-.11	1.02	-.11	.96	-.12
4904	P1, P2, Sim	.55***	.37	.68***	.14	.69***	.21	.71***	.24	.90	-.01	.60***	.28
2484	P1, P2	.55***	.37	.64***	.22	.64***	.27	.69***	.27	.88	.03	.58***	.30
1242	P1	.60***	.33	.66***	.19	.64***	.27	.72***	.24	.97	-.08	.58***	.31
1242	P2	.82**	.07	.84*	-.04	.88*	.00	.89*	.05	.88**	.04	.82*	.01
4423	R. & G. pers.	.55***	.37	.67***	.15	.71***	.19	.75***	.20	.87	.02	.59***	.29
3177	R. pers.	.54***	.38	.66***	.18	.68***	.21	.75***	.20	.81*	.08	.58***	.31
1246	G. pers.	.88*	.01	.82**	-.01	.88*	.00	.97	-.05	1.10	-.21	.87	-.04
													
252	love	.72	.18	.69	.14	.69	.22	.97	-.05	.80	.12	.79	.06
251	values	.96	-.11	.86	-.02	.92	-.04	.96	-.01	.93	.00	.90	-.05
206	sex	.82	.04	.83	.01	.86	.06	.76*	.18	1.00	-.06	.81	.04
280	interests	.99	-.11	1.01	-.21	1.02	-.15	1.02	-.08	1.05	-.16	.95	-.12
245	conflicts	.65**	.25	.70	.18	.74	.18	1.07	-.15	.71*	.20	.67*	.21
182	N-	.70*	.22	.73	.14	.78	.16	1.07	-.13	.71*	.23	.70*	.19
168	O	1.03	-.12	.95	-.15	1.02	-.12	1.04	-.07	1.10	-.19	.96	-.11
392	E	.89	.00	.95	-.12	.96	-.07	1.03	-.12	.97	-.04	.80	.06
238	A	.63**	.29	.69	.18	.70*	.23	1.08	-.15	.71**	.23	.67**	.19
49	C	.94	-.06	.89	-.07	.93	-.04	.99	-.06	1.01	-.08	.93	-.11

Notes. Different sets of variables are included to evaluate their relevance in predicting different RQ measures: Items and scales of actor effects (P1)/ partner effects (P2)/ similarity effects (Sim)

Items and scales of relationship-related personality (R. pers.)/ general personality (G. pers.)

Scales of emotional stability (N-)/ extraversion (E)/ openness (O)/ agreeableness (A)/ conscientiousness (C); Scales of love/sex/conflict/value-related attitudes.

r^2^: forecasting coefficient of determination. Note that, since model training and model evaluation are carried out on different data sets, r^2^ may become negative. MSE: mean squared error.

* p < .05

**p < .01

***p < .001 significantly better than baseline model predicting the RQ average.

To show that our model generation is not affected by overfitting, we conducted the same experiment on a dataset with randomly generated values (see “Supporting information”). We observed an r^2^ close to 0 indicating that our procedure does not suffer from overfitting.

#### 3.2.1. Reproducible predictive power

**Fig 3:**
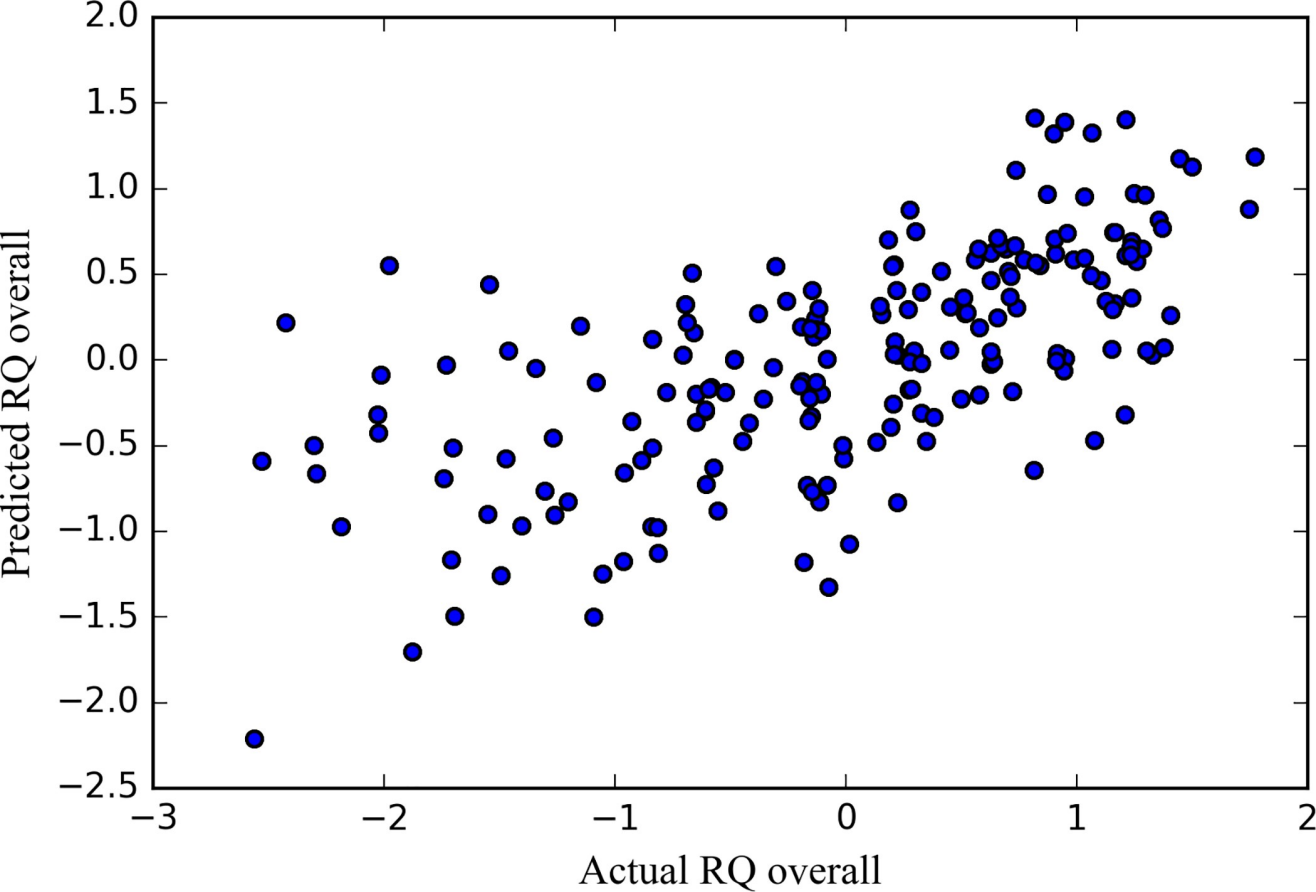
Actual vs. predicted RQ overall for one of the 10 CV iterations based on all actor, partner and similarity variables. Since only the values of one of 10 CV iterations are presented—not the average of all CV iterations: the shown r^2^ and MSE values differ from the performance reported in Table 2. The figure shows that the actual and the predicted outcome are correlated—with the model predicting more accurately on higher values of actual RQ.

The model with all variables could be replicated and explained 37% of the variance of RQ overall in the CV (MSE = .55, r^2^ = .37, p < .001). Fig 3 shows the relation between the predicted and the actual RQ overall values for one of the 10 CV iterations. The visualizations of results of the other 9 CV iterations can be found on our open source page.

Furthermore, the following observations were made regarding the prediction of the different RQ measures:

- Separation intents (MSE = .67, r^2^ = .16, p < .001), partnership satisfaction (MSE = .69, r^2^ = .21, p < .001), sexual satisfaction (MSE = .72, r^2^ = .24, p < .001) and harmony (MSE = .60, r^2^ = .28, p < .001) could be predicted to a similar extent.
- Only ‘Conflicts’ could not be predicted significantly better than the baseline (MSE = .88, r^2^ = .01, p = .172).
- RQ overall could be predicted slightly better than the RQ measures it was generated from.

#### 3.2.2. Actor, partner and similarity effects

Neither partner nor similarity effects predicted incremental variance after accounting for actor features (Plus partner variables: t(99) = 1.57, p = .119. Plus similarities variables: t(99) = .0567, p = .955). Partner variables alone had a slightly lower predictive power compared to actor variables for every RQ measure: e.g. for RQ overall, they significantly differed from one another (t(99) = 3.78, p < .001). Partner variables only explained zero to seven percent of the variance for the RQ measures. Similarity variables did not enhance prediction power (both models: MSE = .55, r^2^ = .37).

#### 3.2.3. Relationship-related and general personality

- Variables of general personality did not have significant predictive power in addition to relationship-related personality variables: while the difference between models based on general vs. general plus relationship-related personality was significant (t(99) = 5.25, p < .001), general personality variables had no relevant effect in addition to relationship-related personality (t(99) = -.553, p = .582). Overall, general personality had a lower predictive power for all RQ measures than relationship-related personality throughout this analysis: e.g. for RQ overall, they significantly differed in their predictive power (t(99) = 5.09, p < .001).
- Models based on conflict-related (MSE = .65, r^2^ = .25, p = .008) variables were more predictive than models based on value-related (MSE = .96, r^2^ < .01, p = .782, n.s.) and interest-related (MSE = .99, r^2^ < .01, p = .948, n.s.) variables. Models based on sex-related (MSE = .82, r^2^ = .04, p = .228, n.s.) and love-related (MSE = .72, r^2^ = .18, p = .063) attributes did not predict significantly better than the baseline.

Models based on variables of agreeableness (MSE = .63, r^2^ = .29, p = .005) and emotional stability (MSE = .70, r^2^ = .22, p = .042) were significantly more predictive than the baseline while models based on variables of conscientiousness (MSE = .94, r^2^ < .01, p = .702, n.s.), extraversion (MSE = .89, r^2^ < .01, p = .403 n.s.) and openness (MSE = .1.03, r^2^ < .01, p = .845, n.s.) were not.

The differences between the model based on conflict-related variables vs. the one based on value-related variables (t(99) = -2.30, p = .023), as well as compared against the one based on interest-related variables (t(99) = -2.50, p = .0142), were significant. Moreover, the models based on openness significantly differed from the one based on emotional stability (t(99) = 2.22, p = .0285), as well as from the one based on agreeableness (t(99) = 3.22, p = .002). In addition, the model based on agreeableness significantly differed from the model based on extraversion at t(99) = -2.05, p = .0431, as well as from the one based on conscientiousness (t(99) = -2.56, p = .0119). Differences between the other models were not significant (t(99)|<2.0, p>.05). Detailed results of all t-tests between the models based on different personality traits can be found on our open source page.

## 4. Discussion

### 4.1. Conclusions

#### 4.1.1. Reproducible predictive power

The ML approach added to the general power and reproducibility of predicting RQ with personality data longitudinally: 37% of the RQ overall measure of couples four years after their personality assessment could be explained using CV. Compared to former studies using simpler correlative analyses with personality data [16, 21], this is a relevant improvement.

The predictive power of the cross-sectional analysis in [26] with a maximum of 24% RQ explained was outperformed indicating that the cross-sectional predictive validity might be different from the longitudinal one. This is in line with the finding of a meta-analysis by Malouff et al. [21], which summarized studies employing simple correlative approaches and showed that the research design (longitudinal or cross-sectional) significantly moderated the effects of personality traits on relationship satisfaction. The indication that RQ at T2 can be better predicted could be due to the fact that T2 RQ has a higher variance: For partners who are still together—as it is the case in cross-sectional analysis—RQ is more homogeneous than in a sample that also includes separated partners. A reason for this might be that partners who are still together idealize the relationship, e.g. because of their feelings of belonging and being part of it, whereas separated partners view their former relationship more realistically or even devalue it to justify the break-up [42, 43].

Follow-up studies could examine whether the RQ of future relationships can also be predicted, especially for break-up as a dichotomous outcome. Other fields in psychology which focus on predicting relevant life outcomes or future decisions with the help of personality traits could also profit from working with ML. Estimating the predictive validity of personality tests with ML could generally contribute to economising them for a specific purpose by only selecting relevant and complementary variables.

#### 4.1.2. Actor, partner and similarity effects

Actor effects alone explained nearly all the variance of the RQ measures, while partner or similarities variables did not have an additional effect. This corresponds with the results of more traditional regression approaches [16]. While actor and partner effects explained variance to a similar extent (18% compared to 27%) when predicting romantic attraction using ML techniques in a small previous study [10], actor effects were more predictive for later RQ in the current study (33% compared to 7% in the cited study): initially being attracted to somebody attractive might more correspond with their characteristics than becoming happy with them later; but both initial attraction, as well as later RQ, might be linked to one’s own traits to a similar extent.

Even the different methods used to scale similarity could not contribute to the power of prediction for RQ. Yet, since this information also is included into the actor and partner variables, they may not have any additional predictive power; another explanation could be that the sample was too small to allow for detecting minor additional effects.

A possible reason why similarities are correlated with RQ might be their correlation with relevant actor effects. It could be the case that similar partners evolve more functional coping strategies with each other or that a functional personality is more likely to look for similar partners. If this were true, solid partner matching would, regardless of the non-additional effect, take the partner similarities into account.

Relationship-satisfaction, sexual satisfaction, separation intents, and harmony could be predicted similarly well by models including actor variables, but these struggled to predict conflicts. By contrast, perception of conflicts seemed not to be linked to actor but by partner effects only. It is possible that conflicts caused by one party are not seen as such by that party; this could be an interesting topic for future work.

#### 4.1.3. Relationship-related and general personality

Replicating former results [20, 26] in the present work, models based on general personality traits predicted RQ less effectively than models based on relationship-related personality traits. Furthermore, as in Großmann et al. [26] general personality had no additional significant predictive power longitudinally when taking relationship-related personality into account. General personality traits might only significantly influence the quality of a partnership when they directly affect interpersonal coping, e.g. are attached to social skills or are experienced in such commitment surroundings as it is the case for agreeableness or neuroticism; both are directly linked to interpersonal conflict coping. While neuroticism includes the tendency to experience negative emotions during conflict, agreeableness contains a set of functional and dysfunctional coping strategies for interpersonal issues and situations. Correspondingly, non-conflict-related attitudes as general values and interests, openness, and conscientiousness do not seem to play a significant role for RQ at all. Even extraversion, which refers to interpersonal contact but not to interpersonal conflict, does not play a major role for RQ.

This way, the present work managed to replicate with data from self-assessment results which had been found with data from behavioural observations [8]: particularly, communication and conflict-related personality characteristics predict break-up and relationship happiness, but not sexual satisfaction. The present work indicates that these characteristics might at least partly be consistent across different relationships. This idea is supported by the finding that questions about the quality of former relationships were among the most important predictors. This general competency in relationship is represented within the love-related and conflict-related variables that reveal to be important for nearly every part of RQ.

### 4.2. Limitations and outlook

In the following sections and in Table 5, the limitations and benefits of the present work are juxtaposed and discussed. In summary, future work should contribute to further improvements in predictions of RQ and to increased generalisability in the models developed.

**Table 5:** Study evaluation.

	Benefits	Limitations
Generalizability	+ Longitudinal design enables prediction over time. + Immanent cross-validation of models protects from overfitting.	- The sample size was restricted. - Only German couples examined. - Only partnerships already existing at T1 were assessed.
Model fit	+ The elastic net with optimization coefficients alpha and lambda could cope with large amounts of highly correlated variables. + Since both partners of a couple always were in either train or test dataset, possible distortions by the nature of dyadic data were eliminated.	- The large numbert of variables in proportion to the sample size restricts model fit. - Only linear effects are analysed. - Only personality traits were used as predictors.
Comparability	+ Models for variable sets and outcomes were systematically juxtaposed.	- The number of variables the models selected from and the number they selected varied.

#### 4.2.1. Sample

Nested CV of models protects from overestimating predictive power and enhances replicability. Nonetheless, the German only sample is a restriction when generalising the results across different cultures. The relatively small sample size also could have limited predictive power, especially due to the comparably high number of variables. Also, since the couples existed at T1, partners of the current sample already influenced one another, e.g. might have changed their partner preferences or their self-perception based on their relationship with the actual romantic partner. This might restrict applicability of the models for partner matching on singles. Although general and relationship-related personality traits turned out to be more robust over time than relationships are [44], it could still be the adaptable, non-stable variance in these trait measurements which are correlated with RQ. To fully ensure applicability in e.g. the dating context, future work has to replicate models in samples of potential partners who get to know each other after they take the personality test.

#### 4.2.2. Study design

Although the current longitudinal design enables prediction over a four-year term, longer-term examinations would still be interesting. An additional strength in terms of comparability is our systematic juxtaposition of models with different variable sets and outcomes. Still, the number of variables, the models selected from, and the number finally selected varied, making a direct comparison between the models difficult. Prediction typically increases in stability with higher numbers of predictors and is therefore more easily significant in comparisons.

Some preceding studies indicated that shared method variance in dyadic data analysis can lead to differences in prediction quality. This has been discussed as a relevant question, especially in the case of partner matching [45]. We solve this issue by assigning the partners of the same dyad both either to the train sample or both to the test sample for every iteration of the CV.

The elastic net managed to cope very well with the large amount of highly correlated variables. Future studies could examine the possibility of unexplained non-linear personality-RQ association, such as those studied by Hudson & Fraley [46] or Joel, Eastwick & Finkel [10] through the application of non-linear ML methods like decision-trees.

Using over 4,000 variables with a wide range of traits and only predicting 37% of the variance means that the scope of the predictive variables we used was limited: it is very likely that there are other variables -beyond personality traits—that could help to achieve a higher predictive power. Therefore, models integrating aspects of the context—e.g. availability and attractiveness of other potential mates or other potentially stressing and protecting factors as standard of living, social support in other relationships and strain at work—could be interesting to further explore the situation-person interaction with the help of ML.

## Supporting information

S1 File Supporting Information. (DOCX)
