# Supplementary material for: Machine learning meets partner matching: Predicting the future relationship quality based on personality traits
Source: PLoS One. 2019 Mar 21;14(3):e0213569. doi: 10.1371/journal.pone.0213569 (PMC6428342; doi:10.1371/journal.pone.0213569)
Supplement: S1 File — (DOCX) [file pone.0213569.s001.docx]

**Supporting information**

To show that the small sample size and the large number of features does result in overfitting of the elastic net model we conducted the following experiment: First, we implemented a scenario where we drew 192 (totally) random instances (cases) of 4904 (random) variables and 192 instances of one additional random “dependent” variable (which due to its random nature, hence, is actually an independent variable).

- 1. We then used a dummy regressor that always predicts the mean value of the target of the training data (this equals the baseline generation in this paper). We observed an average r^2^ of -0.067. Given the random nature of the data and the applied train-test split with 173 vs 19 instances it is reasonable that the train and test mean do differ and thus the prediction must be worse than the test mean, leading to a negative sign.
  2. Next, we fitted an elastic net model on the same data set. We observed a mean prediction r^2^ of -0.104. This value is not significantly worse than the value of the dummy regressor. We conclude that the elastic net does not overfit excessively on randomly generated data even in the light of very few instances and an order of magnitude more variables. Also note that the observed performance is, due to overfitting, worse than the performance of the constant dummy regressor and not better. Consequently, we would expect worse r^2^ values in case of observed overfitting on our train-test split.

We repeated above experiment using the actual RQ as the target variable while all other variables were randomly drown as per our setup above. Also, in this scenario, no overfitting was observed.
